# Retraining automatic action tendencies for smoking using mobile phone-based approach-avoidance bias training: A study protocol for a randomized controlled study

**DOI:** 10.1186/s13063-019-3835-0

**Published:** 2019-12-12

**Authors:** Alla Machulska, Kristian Kleinke, Tanja Joan Eiler, Armin Grünewald, Rainer Brück, Katharina Jahn, Björn Niehaves, Carl Friedrich Gethmann, Tim Klucken

**Affiliations:** 1 Department of Clinical Psychology, University of Siegen, Adolf-Reichwein-Str. 2a, 57068 Siegen, Germany; 2 Department of Medical Informatics und Microsystems Engineering, University of Siegen, Siegen, Germany; 3 Department of Business Informatics, University of Siegen, Siegen, Germany; 4 Research College FoKoS, University of Siegen, Siegen, Germany

**Keywords:** Approach bias, Cognitive bias modification, Cigarette smoking, Nicotine addiction, Randomized controlled trial, Smartphone apps

## Abstract

**Background:**

Automatic tendencies to approach drug-related cues have been linked to the development and maintainance of harmful drug-taking behavior. Recent studies have demonstrated that these automatic approach tendencies can be targeted directly by means of cognitive bias modification (CBM). Moreover, changing those approach tendencies may enhance treatment outcomes. However, training and therapy effects tend to be rather small and adherence to the training might be impaired by time-consuming multiple laboratory training sessions. Here, we present a protocol for a randomized controlled design to improve CBM training efficiency and facilitate access to the training by providing mobile-phone-based training sessions at home to current smokers motivated to quit smoking.

**Methods:**

Participants (*n* = 100) are current smokers who smoke at least six cigarettes per day for at least 6 months and are willing to quit smoking. All participants attend a brief behavioral smoking cessation intervention (TAU) and are randomly assigned either to an experimental (TAU + training) or a control group. Participants in the experimental condition are given access to a training application (app) aimed at retraining automatic approach biases for smoking cues. Participants are instructed to perform the app training outside the laboratory context on a daily basis for 14 consecutive days. Participants in the control group do not receive the training. Primary outcome measures are changes in smoking-related approach biases and reductions in daily nicotine consumption as assessed at baseline, post-training and at 6-week follow up. Secondary outcome measures include approach biases for alternative stimuli or smoking stimuli to which participants were not exposed during training, attentional and association biases, biochemical outcomes, and self-reported smoking behavior, also measured at three different time points (baseline, post-training, and follow up). After completion of the study, smokers in the control condition will receive access to the training app.

**Discussion:**

This randomized controlled trial is the first to test the effectiveness of an app-based CBM intervention as an adjunct to a brief smoking cessation intervention in smokers motivated to quit smoking. The results of this study can inform future research in the optimization and advancement of CBM treatment for addiction.

**Trial registration:**

Current Controlled Trials, ISRCTN15690771. Registered on 20 November 2018.

## Background

Tobacco smoking remains a major public health problem. Smoking is considered the largest preventable risk factor for morbidity and mortality worldwide, with smoking-related deaths estimated at over 5 million people per year [1]. In addition, smoking is strongly implicated in poorer quality of life [2] and imposes a considerably high economic burden on the healthcare system. For instance, it is estimated that smoking-related diseases are responsible for 1.5–6.8% of national health system expenditure [3].

Despite growing evidence about the hazardous effects attributed to smoking, prevalence rates are still high. In Germany, approximately 25–30% of the adult population smoke [4], mostly on a daily basis. Although many smokers want to stop smoking and quit attempts are frequent, relapse rates are upsettingly high [5, 6]. Even following effective treatment, only about one out of four smokers will achieve sustained (≥ 6 months) abstinence [7–9]. Thus, there is an urgent need for developing more effective treatment options and/or improving the efficacy of the available smoking cessation interventions.

While most common interventions for smoking cessation rely on reflective reasoning (i.e. through education on smoking-related health risks, motivational interviewing, and pro–con debates), prominent theories of drug addiction [10, 11] and recent research [12, 13] insist on the notion that more automatic, hard-to-control impulsive processes are strongly implicated in the development and maintenance of addictive behaviors. According to dual-process theories, addiction arises from an imbalance between strengthened impulsive processes at the expanse of weakened reflective processes [10, 11]. Specifically, it is proposed that drug-related cues acquire motivational properties as they predict the availability of drugs, predispose drug taking and/or are associated with rewarding drug effects [14]. Hence, as addiction progresses, information processing is biased in favor of drug-related cues, resulting in various cognitive biases. For instance, research indicates that smokers automatically allocate their attention to smoking-related cues (attentional bias [15, 16]), display implicit positive attitudes toward smoking (association bias; for a review, see [17]), and automatically approach smoking-related cues (approach bias [18, 19]). While all of these biases have been linked to craving, drug seeking and drug use, the approach bias might most strongly relate to actual drug taking, since it incorporates biased information processing with actual motor movement.

Recent research shows that it is not only possible to measure cognitive biases using computer-based tasks, but that such tasks also have merit in modifying existing cognitive biases to promote healthier behavior or abstinence from drug use [12]. In this instance, the Approach-Avoidance Task (AAT) [20] has proven particularly valuable in both measuring and modifying a drug-related approach bias [18, 21]. During the task, different pictures are consecutively presented on a computer screen and participants are instructed to ignore image content and to pull or push a joystick attached to the computer depending on a content-irrelevant feature of the task (i.e., pull all images rotated to the left and push all images rotated to the right). In addition, the AAT incorporates a zoom feature: upon a pull movement, the picture becomes bigger, whereas upon a push movement the picture shrinks, creating a visual sense of approach versus (vs) avoidance. An approach bias is inferred from faster pulling than pushing a picture (e.g., a smoking-related picture). Using the AAT, an approach bias has been associated with different substances, including alcohol [22], cannabis [23], heroin [24], and recently nicotine [18]. Furthermore, an approach bias for cigarette cues has been reported in current smokers, but not in ex-smokers or never-smokers [19]. Lately, the AAT has been adapted to a training variant by changing the contingency between picture content and arm movements, namely by presenting all drug-related pictures in push-away format and all neutral pictures in pull-closer format, thus training automatic avoidance in response to drug-related stimuli. Recently, we applied four sessions of AAT-training as an add-on to a brief smoking cessation intervention in a sample of smokers hospitalized in a psychiatric ward [21]. Compared to sham training where smoking-related pictures had to be pulled and pushed equally often, the AAT-training led to a larger reduction in nicotine consumption at 3-month follow up. In another study conducted by Baird and colleagues [25], smokers motivated to quit were assigned to four sessions of AAT-training or sham training and were asked to make a self-guided quit attempt upon completion of the final training session. Results indicated that the reduction in approach bias was related to the number of days abstinent following the quit attempt. Hence, emerging evidence suggests that cognitive bias modification (CBM) by means of the AAT-training might be a useful intervention to reduce smoking or promote abstinence, but effects tend to be small in size or are even mixed sometimes [26]. Several reasons may account for these heterogeneous findings, with adherence to the demanding training protocols and generalization of training effects among the most significant. For example, although the optimal number of training sessions is still unknown (but see [27], for a systematic investigation on the optimal number of training sessions in the context of alcohol-avoidance training), it seems that training should be administered multiple times to produce stable and long-term improvements. Thus, participants are required to keep multiple training appointments, which begs the question of whether individuals are able to maintain adherence to a daily or weekly training prescription. Indeed, preliminary evidence suggests that high dropout rates are an issue of concern in CBM studies (e.g., [21, 28, 29]). The second reason for rather small training effects concerns the ecological validity of the training tasks. For instance, training takes place in a laboratory context that participants have never before associated with craving, drug taking, or beneficial drug effects. Increasing evidence from the literature on cue exposure and from extinction learning suggest that generalization is inhibited if it takes place in only one context [30–32], leading to reduced treatment effects or increased relapse rates. Thus, CBM efficiency and effectiveness could be improved if the optimal parameters for bias modification are met, including the number and context of training sessions.

One way to improve training adherence and provide varying training contexts could be to apply them via smartphone application (app). An increasing number of people owns smartphones (i.e., 78% in Germany). Moreover, it is estimated, that 3.1 billion people will own a smartphone by the year 2021 [33]. Thus, through smartphone-delivered AAT-training, participants can perform training more often and in the exact same environment in which they otherwise smoke cigarettes, thereby facilitating generalization of training effects.

This study investigates the effectiveness of an app-based AAT-training (app-AAT) for smokers motivated to quit smoking and its effects on the malleability of the smoking-related approach bias, nicotine consumption, and smoking behavior, including cessation. It features a randomized controlled design and multi-session training by means of a newly developed app-based AAT-training during a 2-week training period. We expect that the app-AAT would reduce smoking-related approach biases as measured using the assessment version of the standard AAT [18] and reduce the number of daily smoked cigarettes over and above the control condition.

## Methods/design

### Trial design

The present study is a randomized controlled trial (RCT) comparing self-report, behavioral, and biochemical outcomes of the app-AAT training to a control condition, in which the app training is not received (Additional file 1). Prior to randomization, all smokers included in the study receive standard help for smoking cessation (cf. precise description subsequently). This study employs a 2 (training: training/control) × 3 (time: pretest/posttest/follow up: 6 weeks after baseline) mixed design. The training intervention occurs over the course of 2 weeks in which smokers are given the opportunity to complete AAT-training sessions at home.

### Outcome measures

We focus on two primary outcome measures: (1) changes in smoking-related approach biases with regard to smoking stimuli presented during training (measured in milliseconds) and (2) reductions in self-reported daily cigarette smoking (measured in number of smoked cigarettes per day). Primary time points include post-training and follow-up assessments. Secondary outcome measures are (1) changes in smoking-related approach biases with regard to untrained pictures (close generalization), (2) changes in approach biases for positive pictures, (3) changes in other cognitive biases as measured by the visual dot-probe task and the Implicit Association Task (IAT) (broad generalization), (4) self-monitored smoking behavior using a cigarette tracking app, (5) biochemically verified abstinence rates defined as point prevalence with a 7-day window prior to the assessment point, (6) expired CO, and (7) other self-reported smoking behavior.

### Sample size

A priori power analyses were conducted using G*Power 3.1 (open-source software [34]). Previous research investigating the effectiveness of CBM training identified a small-to-moderate effect size [21, 35]. We conducted a power analysis for a 2 × 3 mixed design ANOVA to detect a small-to-moderate effect (Cohen’s *d* = 0.30, α = 0.05) for the interaction between experimental condition and time for each primary outcome and time point. Power of 0.80 and *r* = 0.5 for correlation between the repeated measures was assumed. Results indicated that 74 participants in total would be needed. Due to an expected attrition rate of approximately 25% at follow up (defined as dropout at any time point after completing the baseline assessment), we decided to include 100 participants, that is 50 per condition.

We would like to stress that we performed power calculations for primary outcomes exclusively. With regard to secondary outcome measures, we emphasize that the study is explorative, meaning that any significant findings on these outcome variables should be interpreted with caution until tested in further confirmatory RCTs.

### Participants

**Fig. 1:**
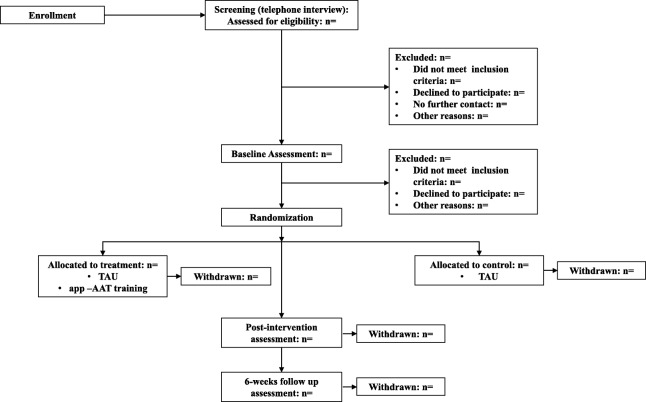
Consolidated standards of reporting trials (CONSORT) flow diagram. APP-AAT, application-based training-Approach-Avoidance Task

Current smokers (*n* = 100) will be recruited at the University of Siegen (Germany) and from the general population. Figure 1 provides the Consolidated standards of reporting trials (CONSORT) diagram of participant recruitment. Participants will be recruited through flyer advertisements, radio broadcasts, television reports, and newspaper advertisement. Interested participants will receive information about the study via e-mail and will be invited to take part in a telephone interview to assess eligibility criteria. Smokers will be included if they have smoked at least six cigarettes per day for at least 6 months. Exclusion criteria are current alcohol or drug misuse or dependency, present psychiatric illness, insufficient German language skills, or uncorrected visual or auditory impairment. Those eligible for the study will be invited for three laboratory sessions. The first two sessions will be 2 weeks apart; the third session will take place approximately 6 weeks after the first (baseline) session. Full written informed consent will be obtained from each participant at study entry (at the beginning of the first session).

### Ethics statement

The study protocol (version 1: 11/2018) was approved by the local Ethics Committee of the University of Siegen (reference number ER_16_2018) and is conducted in accordance with the Declaration of Helsinki and Good Clinical Practice guidelines. During the course of the study, ethical, legal and social aspects (ELSA) will be anticipated and addressed. Each participant will provide written informed consent to participate in the experimental procedure prior to inclusion in our study. Participation will be entirely voluntary and participants will have the right to withdraw their consent for participation at any time.

### Randomization and blinding

Participants will be randomly assigned to the experimental or the control group with a 1:1 allocation ratio, according to an externally constructed randomization plan. To do so, a computer-generated randomization schedule will be employed by means of a computerized random number generator using IBM SPSS Statistics 24. Permuted block randomization will be used to ensure that study groups of approximately the same size are generated. Block size will be set to 50 participants. No other stratification will be used. The study coordinator will perform randomization. Due to the study design, it is not possible to blind participants. However, to avoid bias in the outcome assessment, research assistants concerned with data collection and/or preparation will be blind to the allocation of the participants.

### Intervention

Prior to randomization, participants will receive behavioral counseling and psychoeducation containing information on nicotine addiction and maintenance and short-term and long-term effects associated with cigarette smoking (about 90 min). Afterwards, smokers will be handed a self-help book (a German copy of *The easy way to stop smoking* by Allen Carr) to aid smoking cessation. Finally, participants will be instructed to record cigarettes smoked throughout the day for the entire study period of 6 weeks. Therefore, our behavioral interventions for smoking cessation include brief behavioral counseling, a self-help book, and the instruction to self-monitor smoking. Taken together, these three interventions constitute the TAU condition. Participants allocated to the experimental condition are then given access to the AAT-training app and are instructed to train at least once per day for 14 consecutive days. Participants perform practice training with the researchers to ensure that the training handling and concepts have been fully understood. To ensure compliance, participants will receive daily reminders via short message service (SMS) to complete the training. After completion of the study (after 6-week follow-up assessment), control condition participants will receive access to the training app.

### App-AAT training

The training will be conducted using the AAT-training app, which is based on the AAT-training previously used by our research group [21]. Participants assigned to the training condition will be told that the training is supposed to reduce automatic approach tendencies for cigarette cues. During training, smoking-related or positive pictures appear at the center of the mobile screen. The smoking stimuli consist of pictures of cigarettes or individuals smoking cigarettes. The positive pictures display positive social interactions (i.e., convivial gathering or playing sports) or nature scenes. We decided to use positive stimuli because of the absence of a natural control category for cigarettes. Additionally, a previous study conducted by members of our research group [18, 36] found evidence for a diminished approach tendency for natural reward stimuli in smokers. This reduced sensitivity for rewarding cues might explain why some smokers continue to smoke despite obvious detriments. Thus, we considered that next to reducing maladaptive approach tendencies for smoking cues, it would be valuable to simultaneously increase approach behavior for alternative, positive and non-hazardous activities. Baird et al. [25] kindly provided all pictures. Each of the picture categories contain 25 images presented in a random order. The images are rotated either 3° to the left or 3° to the right. Participants will be instructed to ignore image content and to respond to image orientation by swiping up or down. By swiping up, the picture decreases in size, whereas by swiping down, the picture increases in size, creating a sense of avoiding vs approaching the image. Thus, an indirect instruction is employed. Participants have to execute the correct movement to make the picture disappear. For the purpose of bias assessment, 12 test trials are provided at the beginning of each training session, in which smoking-related and positive pictures appear both in a swipe down and swipe up format. Afterwards, the training starts in which all smoking-related pictures appear in swipe up (avoid) format and all positive pictures appear in swipe down (approach) format. Each picture is shown twice, resulting in 100 training trials per training session. Participants are allowed to take a short break halfway through. Training sessions take approximately 10 min to complete.

To access the training, each participant is required to enter his/her personalized participant-ID before he/she can log on to the app. After completion of each training session, a training file including participant-ID, date of the training sessions, and reaction times (RTs) in milliseconds per trial will be uploaded to a server enabling study investigators to download and view the file. In doing so, we can track the number of training sessions completed by each participant and thereby assess fidelity.

### Material and measures

#### Cognitive bias assessment

To investigate training effects and to test for generalization of training effects, three different cognitive biases will be assessed at baseline, post-training, and follow up.

##### Approach bias assessment

Automatic approach biases for smoking-related stimuli will be measured by means of the standard version of the nicotine-AAT (see [18]). At baseline, 25 smoking-related and 25 positive images derived from Baird et al. [25] appear on a computer screen. To test whether training effects can be generalized to pictures other than those used during bias retraining, 12 out of the 25 pictures contain images not shown during app-AAT training (close generalization). Similar to the training, images are tilted 3° to the left or 3° to the right. A joystick (Logitech Extreme 3D) is connected to the computer and participants are told to use the joystick to push images rotated to the right and to pull images rotated to the left. As a result, images shrink or grow in size dependent on arm movement. For the purpose of bias assessment, smoking and control pictures have to be pulled and pushed equally often. Each picture is shown once in push-away format und once in pull-closer format, resulting in 100 assessment trials.

An approach bias in inferred from faster picture pulling than picture pushing. That is, an approach bias score is calculated by subtracting median RTs for pulling a picture from median RTs for pushing the exact same picture. The RT is defined as the time a participant needed to execute the correct full joystick movement. Accordingly, a positive value indicates an approach tendency toward a picture category, whereas a negative value indicates an avoidance tendency. Approach biases are computed for each of the image categories (smoking vs positive; trained vs untrained) and for each of the laboratory sessions (baseline, post-training, and follow up), resulting in four bias scores at three different time points.

##### Attentional bias assessment

Automatic attentional biases for smoking cues will be measured by a visual dot-probe task adapted from Miller and Fillmore [37]. The task will be operated using Inquisit Lab software and will be performed on a personal computer. Each trial starts with a 500-ms fixation cross in the center of the computer screen. Afterwards, two 13 × 18 cm pictures (a smoking-related and a control picture) appear side by side, 3 cm apart. The position of the pictures is randomly chosen as either left or right to the location of the fixation cross. After a short duration of 1000 ms, the two pictures disappear and a probe stimulus (here “X”) appears in the location of one of the pictures. A response pad (Cedrus Response Pad RB844) is attached to the computer and participants are asked to press a yellow key if the probe is left and to press a green key if the probe is right. Note that we decided to use response pads instead of serial keyboards since subject responses with standard PC keyboards are frequently associated with accuracy problems. As keyboards are not generally designed to be fast input devices in absolute terms, RTs can vary when using serial keyboards due to polling loops and the electronics in the keyboard. For instance, Plant and Turner [38] compared four different keyboards and found that on average, 18–34 ms were added to response times with different amounts of variability. Such timing errors are crucial given that cognitive bias assessment relies on rather small RT differences (on average 40–80 ms on a group level). Furthermore, accuracy problems in cognitive bias assessment might account for null findings, replication failure, or inconsistencies in the literature [39, 40].

The task stimuli consist of 10 smoking-related images that were matched with 10 neutral tooth-cleaning control images. This image set was kindly provided by Stippekohl and colleagues [41] and has been previously used in other studies conducted by members of our research group [18, 21, 36]. One major advantage of this picture set is that smoking and control pictures were carefully matched in terms of colors and shape (see [41]). In addition, contrary to the pictures used in the AAT, which display complex scenes, these pictures contain a simple smoking or tooth-cleaning related content without distractors. This fact is crucial since evidence hints to the fact that complex addiction-related scenes might be less effective at capturing participants’ attention and could therefore result in less attentional bias when used in visual probe tasks [37]. Each image pair is presented four times, resulting in 40 test trials. In addition, 40 filler trials are included, which consisted of 10 pairs of neutral images. This is done to reduce possible habituation to smoking-related stimuli that might occur otherwise [42]. Test and filler trials are presented randomly, resulting in 80 trials in total. Filler trials are not included in the final data analysis.

An attentional bias is inferred from faster responding to probes replacing a smoking-related image than to those replacing an image unrelated to smoking. To calculate an attentional bias score, RTs for probes replacing smoking pictures are subtracted from RTs for probes replacing tooth-cleaning pictures. Thus, a single measurement of attention bias at each of the three assessment times emerges. A positive value reflects an attentional bias toward smoking-related pictures while a negative value is indicative of an attention bias for tooth-cleaning pictures.

##### Association bias assessment

Implicit positive or negative associations for smoking will be measured by an implicit association task (IAT) [43]. The task was adapted from Kahler and colleagues [44]. Participants are asked to categorize positive and negative attributes (e.g., “welcomed” vs “disrespected”) and target items (e.g., pictures of a cigarette vs a chair) into predetermined categories via response pad button presses (Cedrus Response Pad RB844).

Following Greenwald et al. [45], the IAT is organized in seven blocks: (a) a 24-trial target discrimination block (e.g., press yellow for “smoking” vs press green for “furniture”); (b) a 24-trial attribute discrimination block (e.g., yellow for “I feel positive” vs green for “I feel negative”); (c) a 24-trial practice combined block (e.g., yellow for “smoking” or “I feel positive” vs green for “furniture” or “I feel negative”); (d) a 40-trial test combined block (same as practice); (e) a 24-trial target discrimination block, in which the target categories are reversed (e.g., yellow for “furniture” vs green for “smoking”); (f) a 24-practice combined block with reversed target categories (e.g., yellow for “furniture” or “I feel positive” vs green for “smoking” or “I feel negative”), and (g) a 40-trial test combined block (same as practice). Blocks c, d, f, and g are crucial blocks used in scoring the IAT.

Assuming that participants associate smoking with positive feelings, trials in which smoking and positive consequences share a response key are congruent, whereas trials in which smoking and negative consequences share a response key are incongruent. The IAT score will be calculated by subtracting RTs for incongruent blocks (i.e., smoking + negative; furniture + positive) from RTs for congruent blocks (i.e., smoking + positive; furniture + negative). Larger IAT scores suggest stronger implicit, positive, social associations with smoking. Similar to the attention bias calculation, a single IAT score results for each of the assessment times.

To prevent methodological confounds, the IAT is counterbalanced in two ways: first, the placements of the target and attribute stimuli labels are counterbalanced (yellow or green button). Second, two IAT orders are used: one with the congruent combination block first and one with the incongruent combination block first. The two IAT orders will be counterbalanced across participants.

### Biochemical verification

Expired CO will be assessed at baseline, post-training, and at 6-week follow up using a Carbon Monoxide Monitor (piCO™ Smokerlyzer®; Bedfont Scientific Ltd).

### Behavioral and self-report measures

To track daily smoking and possible changes in nicotine consumption, participants are asked to self-monitor their smoking behavior via a cigarette-tracking app, which was specifically designed for the purpose of this study. That is, participants are required to download a native journaling application and are instructed to log cigarettes during or directly after smoking. The data will then be charted on the phone and on the companion website to which it is automatically synchronized. In addition, participants will be asked to estimate their average daily nicotine consumption at pretest, post-training and follow up.

The questionnaire measures will include the Fagerström Test for Nicotine Dependence (FTND) [46] (German version: [47]), the Stages of Change Scale [48] (German version: [49]), the Thoughts About Abstinence Scale [50], attitudes toward smoking based on Swanson et al. [51], the Barratt Impulsiveness Scale (BIS) [52], and the Eysenck Personality Questionnaire - Revised (EPQ-R) [53]. In addition, participants indicate their level of cigarette craving on a 6-point Likert-scale ranging from 0 (“not at all”) to 5 (“very high”). Questionnaire measures will be used to examine group differences at baseline and to assess any changes across the course of the study. At posttest, participants in the experimental condition will be also required to evaluate the app-AAT training and indicate their awareness about the training contingency.

### Procedure

**Fig. 2:**
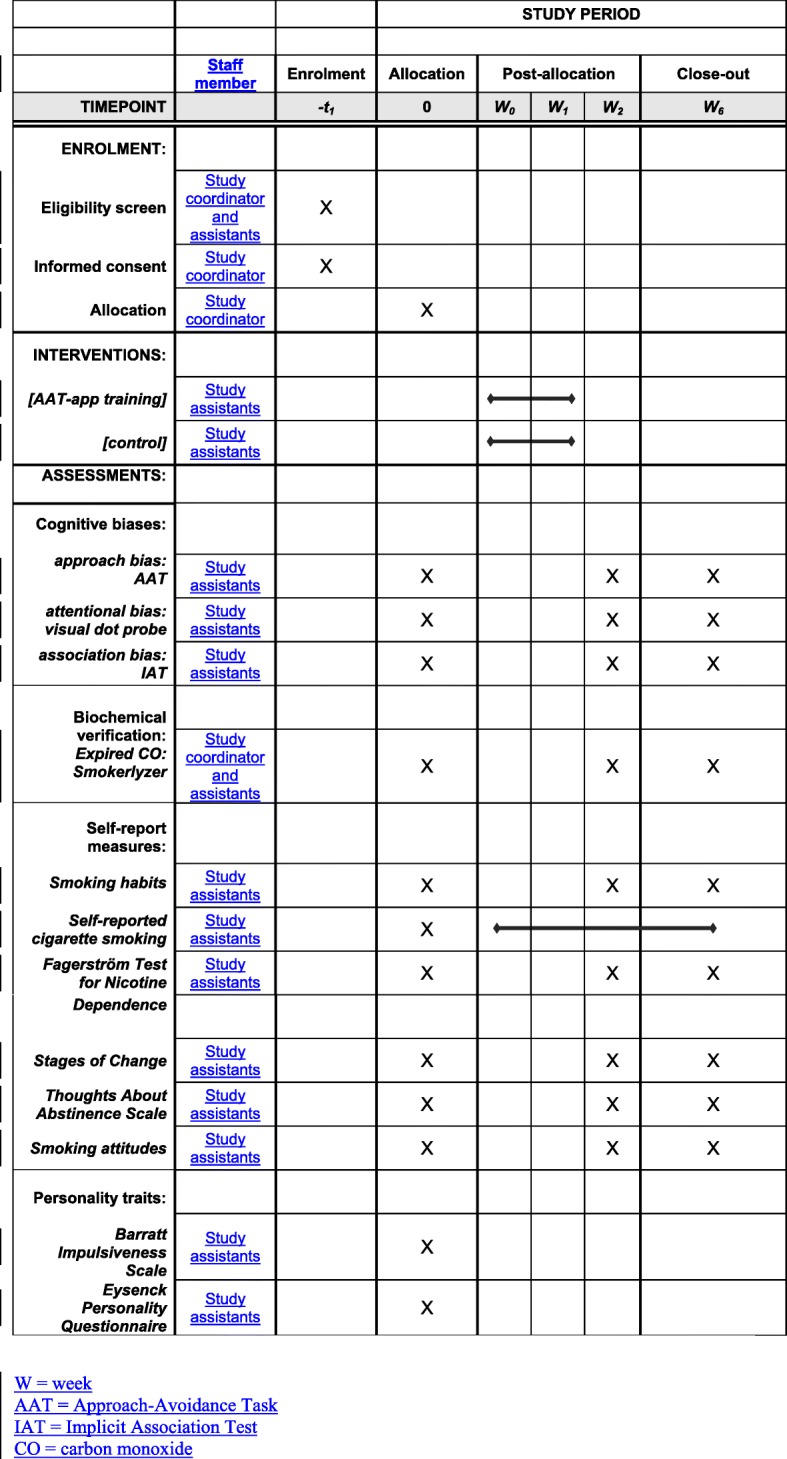
Schedule of enrollment, assessments and interventions (SPIRIT)

A time schedule of enrollment, assessment visits, and AAT-app trainings for participants is shown in Fig. 2. During the first laboratory session (baseline; around 180 min) participants give informed consent and take part in a brief behavioral intervention for smoking cessation (TAU). Participants then complete the cognitive bias assessments, the questionnaire measures, and the carbon monoxide breath test. Smokers in the training condition (TAU + training) are given access to the AAT-training app. Participants are instructed to perform the app training on a daily basis for 14 consecutive days. During the second (post-training) and final (6-week follow up) laboratory session, participants complete cognitive bias assessment tasks, questionnaire measures, and the CO breath test. Participants in the experimental condition are asked to evaluate the training and indicate their awareness about training contingencies. Individuals randomized to the control condition will receive access to the training app upon completion of the final follow-up measure. Training data from these participants will not be analyzed.

### Data preparation and planned analyses

Multiple imputation of missing data will be performed for intention-to-treat (ITT) analyses [54]. To analyze changes in cognitive biases, error trials will be excluded from further analyses. Median RTs will be used to minimize the influence of outliers.

To investigate whether the app training leads to changes in the primary outcome measures, two separate 2 (condition: training vs control) × 3 (time: baseline, post-training, follow up) mixed design analysis of variance (ANOVA) will be performed. Changes in smoking approach biases and self-reported daily nicotine consumption will constitute the primary dependent variables. A separate univariate test will determine if there are significant differences in smoking-related approach biases or average nicotine consumption at baseline between the app training and the control group. Due to the random assignment to the experimental or to the control group, we expect a priori group differences to be small. Nevertheless, chance baseline imbalance between treatment arms will be taken into account, when evaluating training effects. Substantive hypotheses will be tested by means of follow-up simple effects analyses. Here, we compare means in the app training and control group (posttest and follow up, respectively), while controlling for baseline approach bias and nicotine consumption levels. We apply a mixed design ANOVA as one factor (a fixed effects factor) is a between-subjects variable (here, condition) and the other (a random effects factor) is a within-subjects variable (here, time).

To test whether the app training leads to changes in the secondary outcome measures, parametric and non-parametric statistical tests will be administered where appropriate. Close training generalization will be investigated via a 2 (condition: app-training vs control) × 2 (image content: smoking vs positive pictures) × 2 (generalization: trained vs untrained pictures) × 3 (time: baseline, posttest, follow up) mixed-design ANOVA. The 2 (condition) × 3 (time) mixed-design ANOVA will be performed separately for attentional and association biases. Multilevel modeling (MLM) will be used to estimate the growth curve for nicotine consumption as measured with the cigarette training app over time (throughout the entire study period from baseline to follow up). Time will be modeled as a continuous variable. The major advantage of MLM is that it increases power and generalizability by including all individuals regardless of missing data. To examine whether the app-AAT training leads to increased abstinence rates, for each following time point, a separate chi-square test will be carried out with the binary outcome variable (smoking vs nonsmoking) as the parameter of interest. To test the effect of training on other smoking-related variables (i.e., CO, craving, FTND, smoking attitude, motivation to quit), a 2 (condition) × 3 (time) repeated measures multivariate ANOVA (MANOVA) will be conducted. MANOVA with a significant result will be followed up with both univariate tests and discriminant factor analysis. That is, ANOVA will be carried out separately on each of the dependent variables. These analyses will in turn be followed up using contrasts. In addition, discriminant analyses will be applied, which take linear combinations of the dependent variables into account, thereby complementing univariate approaches.

## Discussion

This study makes use of a randomized controlled design to explore the efficacy of an app-based approach bias retraining in a sample of regular smokers motivated to quit smoking. We seek to investigate whether our newly developed app-AAT training will modify cognitive biases, reduce smoking behavior and contribute to quit attempts and smoking cessation. To do so, an approach bias retraining app will be added to “treatment as usual”, which comprises information about smoking, a self-help book for smoking cessation, and self-monitoring daily cigarette use.

Recent work on CBM in the context of addiction and unhealthy behavior shows promise in changing maladaptive cognitive biases and/or reducing harmful behavior [12, 55]. However, several challenges have to be addressed, including small-to-medium training effects, adherence to the training, and generalization to long-term, real-world behavior. Providing the opportunity for participants to perform training via mobile phones at home represents a promising innovation in the field of CBM research, not only because the majority of people possesses smartphones, but also because individuals tend to keep their mobile phones close to hand in nearly any everyday situation. Hence, next to their ubiquity, smartphones can provide novel ways to optimize training efficacy and efficiency and to increase the number of opportunities to engage in training. This proof-of-principle study is novel in combining existing approach bias retraining for smoking with a mobile-phone-based application as an adjunct to brief behavioral counseling for smoking cessation. Our results can inform future research and clinical practice, as CBM could provide a cost-effective and easy-to-access intervention that could be used as an add-on to more traditional treatments, to promote abstinence from smoking or other harmful activities.

## Trial status

At the time of submission, the trial had not started. Recruitment is scheduled to begin in November 2019. The expected duration of the study is 12 months. The final results will be published as soon as possible after the analysis is completed.

## Supplementary information

**Additional file 1.** SPIRIT 2013 Checklist: Recommended items to address in a clinical trial protocol and related documents*.

## Data Availability

The datasets used and/or analyzed during the current study are available from the corresponding author on reasonable request.

## Abbreviations

AAT: Approach-Avoidance Task
ANOVA: Analyses of variance
App: Application
BIS: Barratt Impulsiveness Scale
CBM: Cognitive bias modification
CO: Carbon monoxide
CONSORT: Consolidated standards of reporting trials
ELSA: Ethical legal and social aspects
EPQ: Eysenck Personality Questionnaire
FTND: Fagerstörm Test for Nicotine Dependence
IAT: Implicit Association Task
ITT: Intention to treat
MANOVA: Multivariate analysis of variance
MLM: Multilevel modeling
Ms: Millisecond
RCT: Randomized controlled trial
RT: Reaction time
SMS: Short message service
TAU: Treatment as usual
